# Radiomics applications in cardiac imaging: a comprehensive review

**DOI:** 10.1007/s11547-023-01658-x

**Published:** 2023-06-16

**Authors:** Tiziano Polidori, Domenico De Santis, Carlotta Rucci, Giuseppe Tremamunno, Giulia Piccinni, Luca Pugliese, Marta Zerunian, Gisella Guido, Francesco Pucciarelli, Benedetta Bracci, Michela Polici, Andrea Laghi, Damiano Caruso

**Affiliations:** Department of Medical Surgical Sciences and Translational Medicine, Sapienza University of Rome - Radiology Unit - Sant’Andrea University Hospital, Via di Grottarossa, 1035-1039, 00189 Rome, Italy

**Keywords:** Radiomics, Texture analysis (TA), Coronary computed tomography angiography (CCTA), Cardiac magnetic resonance (CMR), Ischemic heart disease (IHD), Hypertrophic cardiomyopathy (HCM)

## Abstract

Radiomics is a new emerging field that includes extraction of metrics and quantification of so-called radiomic features from medical images. The growing importance of radiomics applied to oncology in improving diagnosis, cancer staging and grading, and improved personalized treatment, has been well established; yet, this new analysis technique has still few applications in cardiovascular imaging. Several studies have shown promising results describing how radiomics principles could improve the diagnostic accuracy of coronary computed tomography angiography (CCTA) and magnetic resonance imaging (MRI) in diagnosis, risk stratification, and follow-up of patients with coronary heart disease (CAD), ischemic heart disease (IHD), hypertrophic cardiomyopathy (HCM), hypertensive heart disease (HHD), and many other cardiovascular diseases. Such quantitative approach could be useful to overcome the main limitations of CCTA and MRI in the evaluation of cardiovascular diseases, such as readers’ subjectiveness and lack of repeatability. Moreover, this new discipline could potentially overcome some technical problems, namely the need of contrast administration or invasive examinations. Despite such advantages, radiomics is still not applied in clinical routine, due to lack of standardized parameters acquisition, inconsistent radiomic methods, lack of external validation, and different knowledge and experience among the readers. The purpose of this manuscript is to provide a recent update on the status of radiomics clinical applications in cardiovascular imaging.

**Supplementary Information:**

The online version contains supplementary material available at 10.1007/s11547-023-01658-x.

## Introduction

Over the past two decades, the utility of coronary computed tomography angiography (CCTA) and cardiac magnetic resonance (CMR) as diagnostic technique for improving patient care in cardiovascular medicine has been well established [1, 2]. In consideration of its high diagnostic performances, CCTA has been recently included in the international guidelines of the European Society of Cardiology (ESC) as a Class I recommendation for diagnosis and management of chronic coronary syndromes [3]. CMR has become a valuable tool in diagnosis, risk stratification, and follow-up of patients with ischemic heart disease (IHD), cardiomyopathy, myocarditis, and in the evaluation many others structural cardiac diseases [4].

Currently, advances in the field of cardiac imaging have made possible to define and validate cardiac damage markers, such as late gadolinium or iodine enhancement. However, the reader’s experience plays a determinant role in visual evaluation of CMR and CCTA, and therefore the ability to discern subtle differences or to define new imaging markers. New image analysis techniques, based on quantitative evaluation of data extracted from radiological images, can improve diagnostic and prognostic accuracy, decreasing readers’ visual biases and subjectivity [5].

In the new era of “personalized therapy” and “precision medicine,” many emerging translational fields of research, such as genomics, proteomics, or metabolomics, raised up to support evidence-based clinical decision making [6, 7]. In a similar way to these widely known “omics” sciences, a rapidly evolving field of research, called “radiomics,” was born from the increasing use of medical imaging [8].

Radiomics is a promising field of medical research consisting in the extraction and analysis of quantitative metrics from medical images, resulting in a conversion of images into mineable data [8, 9]. The primary assumption behind radiomics is that a wide range of information, imperceptible by the human eye and inaccessible through traditional qualitative visual analysis, are contained in biomedical images [10]. Thus, the aim of radiomics is to obtain from hidden insights new noninvasive imaging biomarker, able to correlate with prognosis and treatment response [11].

The aim of this review is to provide an overview of radiomic technical principles and to describe the clinical applications in cardiovascular imaging and the prominent results focusing on CCTA and CMR.

## Technical principles

**Fig. 1:**
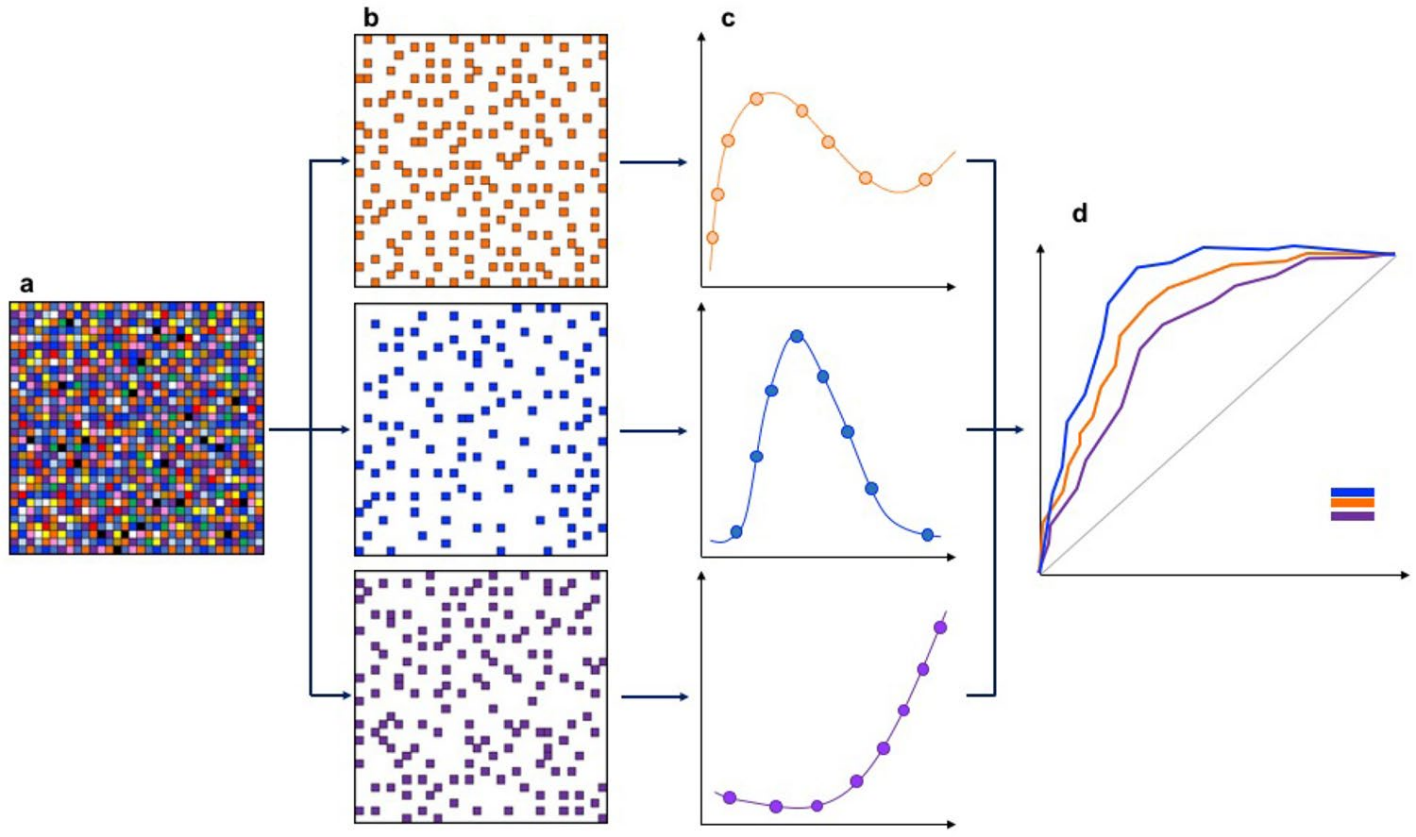
Schematic representation of radiomics workflow, **a** Images segmentation: in each manually or automatically drawn ROI can be found a large amount of data, impossible to detect by visual assessment only, that can be analyzed with sophisticated Radiomics software. **b** Dataset split and features extraction: data were extracted separately and sorted, to be analyzed as independent variable. **c** Statistical analysis: each variable underwent statistical analysis in order to find best performing parameters. **d** Model construction and validation: most effective variables were utilized to build prediction models that were tested with ROC (receiver operating characteristic) curve analysis; valuable models should be applied to internal or external cohort to be definitely validated

Radiomics workflow is divided into subsequent and interdependent steps: images acquisition, raw data reconstruction, images pre-processing, image segmentation, features extraction, features selection, model construction, and validation (Fig. 1) [12].

Imaging acquisition is probably the most challenging step, since the lack of standardized acquisition protocols affects analysis reproducibility. Therefore, the implementation of standardized data acquisition protocols and data analysis techniques is of paramount importance to provide a solid framework for radiomic analysis [13, 14].

Image segmentation is the second step of radiomic workflow, in which one or more ROIs are drawn to encompass the anatomical region to be analyzed [15]. Different segmentation systems are possible: manual, semi-automatic, or fully automatic. Manual segmentation is a time-consuming process relying on the radiologist’s experience and is affected by broad intra-observer and inter-observer variability. On the other way, semi-automatic and automatic segmentation systems have shown promising results due to high accuracy, low inter-reader variability, and high reproducibility.

In field of radiomic workflow automation, the application of artificial intelligence is proving to have a potential role in overcoming some limitations, such as the long lead times and the slow process of manual analysis [16, 17].

Once images have been segmented, quantitative parameters are extracted from previously outlined ROIs; the obtained features are divided into *shape features*, describing the shape and geometry of the ROIs (e.g., volume, maximum surface): *first-order*, *second-order*, and *higher-order* statistics [12].

First-order statistics are also referred to as intensity-based and assess voxel distributions without considering their spatial relationship, including kurtosis, skewness, first-order entropy, mean of all pixel, mean of positive pixels, and standard deviation [13].

Second-order statistics takes into account spatial relationships among adjacent voxels [7], including run-length matrix (RLM), gray-level co-occurrence matrix (GLCM), and gray-level run-length matrix (GLRLM). Higher-order statistics analyze the relationships and differences of multiple pixels after applying filters or mathematical transforms to the images [9].

Once features are extracted, the next steps of the radiomic workflow consist of designing statistical models in which the most performing image features are combined with clinical data to provide a tool capable of supporting clinical decision making. Several statistical methods are usually applied to build such models, such linear regression, logistic regression, Cox proportional hazards regression, least absolute shrinkage, and selection operator (LASSO) [18]. Once radiomics models are built, they must be optimized and validated, in order to optimize their clinical reliability [12].

Texture-based metrics have been widely used in oncology to improve diagnostic accuracy, staging, grading, and response to treatment. Nevertheless, such novel technique has been scarcely applied in cardiac imaging, probably due to the complexity of cardiovascular imaging techniques [14].

This new discipline has the potential to increase our medical knowledge and ensure greater efficacy of clinical decisions.

## Radiomics application in coronary computed tomography angiography (CCTA)

CCTA should be considered in selected patients, stratified by clinical likelihood, as the first-line diagnostic test in chronic coronary syndromes, as well as other noninvasive techniques and invasive coronary angiography (ICA), according to ESC 2019 guidelines [3], due to its ability to rule out coronary stenosis with a NPV of 97–99%, and to its direct visualization of vessel wall and plaque morphology [15, 19].

Radiomics applied to CCTA consists in building big-data repositories in which each lesion is characterized by hundreds of different parameters impossible to detect by mere visual assessment, thereby allowing the improvement of post-processing analysis and interpretation of radiological images [20]. The aim of this section is to review the current radiomics applications in cardiovascular CT.

### Radiomics in CCTA evaluation of coronary plaques vulnerability

Plaque rupture with subsequent intraluminal thrombosis is the most common cause of acute myocardial infarction (AMI) [21]. Therefore, one of the main challenges of modern cardiac radiology consists in identifying reliable markers of plaque instability, aiming at preventing major ischemic events.

Four distinct plaque characteristics derived from CCTA have been linked to major adverse cardiovascular events: positive remodeling, low attenuation, spotty calcification, and napkin-ring sign (NRS) [1, 22]. The latter representing the only non-quantitative marker, depending on readers’ visual interpretation. NRS, described as a plaque cross section characterized by a core area of low CT attenuation surrounded by a thin rim of higher CT attenuation [23], may predict the ischemic burden of a plaque independently from the grade of stenosis [24]. Several studies have shown that radiomics could strengthen the identification of NRS by an objective assessment of CT images.

**Fig. 2:**
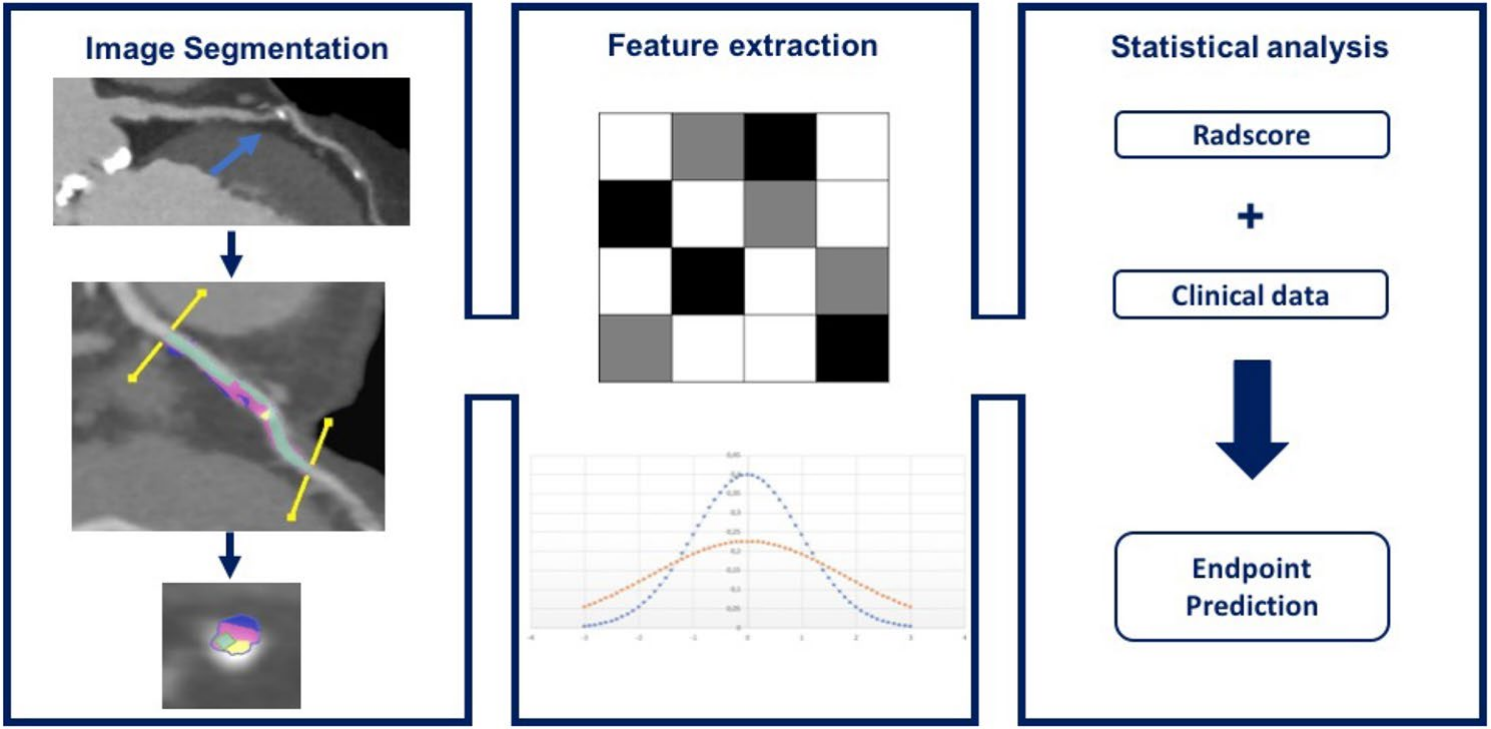
Low-attenuation plaque CCTA radiomics workflow. Image segmentation: low-attenuation plaque (blue arrow) semi-automatically segmented on curved reconstructions and on axial section. Feature extraction: low-attenuation plaque texture and integration data. Statistical analysis: combination of radiomic features with clinical data to obtain predictive model. Cardiac computed tomography angiography (CCTA)

In a retrospective analysis, Kolossváry et al. [25] revealed the high effectiveness of radiomics analysis in the identification of plaques with NRS. Authors enrolled 2674 consecutive patients who had undergone CCTA for stable chest pain, identifying 30 NRS plaques in 30 patients (NRS group) and a non-NRS control group with matching demographics. After manual plaque selection, image segmentation and data extraction were performed using an automated plaque analysis tool (Fig. 2). For each coronary plaque, a total of 4440 radiomics features were extracted, of which 916 (20.6%) provided a significant difference between the two groups. Four hundred forty features (9.9%) had an area under the curve (AUC) > 0.80: in detail, 8 parameters among first-order statistics features, 348 parameters from GLCM, 30 parameters from GLRLM, and 54 parameters from geometry-based features. Furthermore, no significant differences between groups in traditional plaque characteristics have been found.

These results demonstrated that radiomics analysis on coronary plaques is possible and effective, reaching greater diagnostic performance than conventional evaluation. Results also revealed that voxels’ spatial distribution parameters (GLCM, GLRLM, and geometry-based) had a better predictive value as imaging markers of NRS detection than first-order statistics.

CCTA data should be integrated with data from other imaging modalities in order to fully understand the evolution of CAD and to prevent an adverse event. Invasive coronary angiography is characterized by great spatial resolution and detailed vessel lumen depiction, yet fails to visualize the vessel wall [26]. In contrast, intravascular ultrasound (IVUS) and optical coherence tomography (OCT) allow direct plaque visualization and characterization [27]. In particular, IVUS identifies soft plaques as hypoechoic, while OCT identifies thin-cap fibroatheromas. Nevertheless, invasiveness and low accessibility limits the widespread application of these imaging modalities.

On the other side, combined PET/CT merge anatomical and functional information [15, 28]: in patients with stable CAD, sodium fluoride positron emission tomography (NaF^18^-PET) identifies inflammation and microcalcifications in coronary atherosclerotic plaques, widely accepted hallmarks of plaque metabolic activity and vulnerability [15, 29]; the main limitations of PET/CT are radiation exposure.

Since each modality has one or more limitations and cannot assess the entire spectrum of plaque characteristics, a noninvasive imaging modality able to identify alternative biomarkers of plaque vulnerability would be highly desirable; again, radiomics may be a suitable and effective candidate.

A recent investigation [30] analyzed 44 plaques in 25 patients who had undergone CCTA, NaF^18^-PET, IVUS, and OCT, aiming at assessing the reliability of CCTA radiomics features in finding new markers of plaque vulnerability. Among the extracted radiomic features, *fractal box counting dimension of high attenuation voxels* showed good diagnostic accuracy to identify IVUS-attenuated plaques (AUC 0.72) and excellent diagnostic accuracy to identify OCT thin-cap fibroatheromas. (AUC 0.80). Fractal dimensions are related to the spatial complexity of structures: it quantifies the changes of object details in relation to variations of scale [14]. High-risk plaques presented the low-attenuation voxels of the lipid pool in their central portion, next to each other, while higher attenuation voxels are relatively scarce and occupy limited space: therefore, these plaques return low values of *fractal box counting dimensions of high attenuation voxels*. On the other hand, stable plaques have scattered higher attenuation voxels, resulting in higher values of that radiomic feature.

Although CCTA is an anatomical and non-functional imaging modality, this research also showed that radiomics can efficiently identify NaF^18^-PET positive plaques, characterized by inflammation and microcalcifications. The *surface of high attenuation voxels* parameter reached the best diagnostic accuracy (AUC 0.87) in identifying plaques with increased radionuclide uptake.

In conclusion, these results suggested that radiomics principles applied to CCTA may represent a noninvasive alternative to IVUS, OCT, and PET/CT in identifying with good-to-excellent diagnostic accuracy markers of plaque vulnerability, outperforming conventional CT metrics.

### Radiomics in CCTA evaluation of advanced atherosclerotic lesions

Advanced atherosclerotic lesions, like early and late fibroatheromas and thin-cap fibroatheromas, are frequently associated with a higher risk of myocardial infarction [31];Moreover, low-attenuation burden has recently been shown to be the strongest predictor of the evolution of the atherosclerotic disease and of fatal or non-fatal myocardial infarction [32, 33].

Conventional CCTA classification, which divides plaques in calcified, partially calcified, and noncalcified, is outperformed by the *plaque attenuation pattern scheme*, a model able to differentiate atherosclerotic coronary plaques into homogeneous, heterogeneous, and with NRS. Both classification systems are visual and have several limitations, above all the lack of reproducibility, even among experienced readers [34]. A quantitative system, named *histogram-based method*, was developed aiming at overcoming visual analysis limitations, demonstrating that density values < 60 HU accurately identify lipid-core plaques [35]; however, the reliability of this method is hampered by the effect of different tissue components on UH values [36].

A recent investigation [37] demonstrated that machine learning-aided radiomic analysis outperforms conventional and histogram-based CCTA methods in distinguishing between early and advanced histological confirmed atherosclerotic lesions. In particular, among the different models tested, the *least angles regression* model provided the best discriminatory power. Thus, radiomics-based ML technique improves the diagnostic power of CCTA and outperforms expert conventional visual assessment and histogram-based methods in the identification of advanced atherosclerotic lesions.

### Radiomics in CCTA evaluation of pericoronary adipose tissue (PCAT)

The pericoronary adipose tissue (PCAT) influences the local vascular biology through a bidirectional interaction with the coronary wall: adipocytes release the adipokines, active molecules that exert paracrine effects on the vascular wall, contributing to the local inflammation reaction, which plays a key role in atherogenesis. Therefore, quantitative and qualitative evaluations of PCAT are currently considered critical in classifying individual cardiometabolic risk profile [38].

Radiomics could be a useful tool for an objective analysis of PCAT inflammation-induced permanent changes and, indirectly, of chronic coronary inflammation. In this regard, in a recent study of Oikonomou et al. [39] three different and independent potentially relevant correlations with PCAT were examined.

The first part of the study evaluated the correlation between fat biology and radiomic features, including 167 patients who underwent CCTA and PCAT sampling during cardiac surgery. Results revealed that *wavelet-transformed mean attenuation*, a first-order feature, was the best performing metric for PCAT inflammation detection, as confirmed by the relative expression of TNFA. On the other side, higher-order statistics features showed better performances in detection of adipose tissue fibrosis and vascularity than the simple mean attenuation, as confirmed by the relative expression of COL1A1 (alpha 1 chain of type-1 collagen) and the endothelial marker CD31 (platelet endothelial cell adhesion molecule-1).

In part two, a pool of 5487 CCTA included in the CRISP-CT [40] & SCOTHEART [41] studies were retrospectively analyzed and 1391 stable radiomic features were extracted, in order to develop and validate the pericoronary fat radiomic profile (FRP), as predictive factor of major adverse cardiac events (MACE). 101 MACE cases and 101 MACE-free cases were matched, training and validating a random forest algorithm to discriminate cases from controls, and sixty-four features were eventually selected, representing the definitive radiomics substrate of FRP. FRP then was positively associated with risk MACE with a cutoff point of 0.63. In summary, FRP improved the performances of the traditional model and has been proven to be an independent predictor of MACE.

Finally, the third part of the study included 88 patients (44 patients with AMI and 44 stable CAD controls) and assessed the ability of FRP in detection of unstable coronary plaques and changes over 6 months post-AMI. Results showed that the FRP was significantly higher in AMI group, without any modification changes over 6 months post-AMI, suggesting one more time its potential role in detection of irreversible changes, such as vascular alterations and fibrosis of adipose tissue. In contrast, the perivascular FAI (Fat Attenuation Index) measured around the right coronary artery (RCA) changed dynamically at 6 months, demonstrating that it could be considered a specific biomarker of the overall reversible and dynamic changes due to coronary inflammation.

Surprising results of TA applied to PCAT were also reached by Lin et al. [42] that compared 60 patients with AMI who underwent CCTA within 48 h of admission, 60 patients with stable CAD, and 60 controls with no CAD. PCAT was segmented around culprit and non-culprit lesions in patients with AMI and around the proximal RCA in all patients. Results demonstrated that patients with AMI were characterized by a distinct PCAT radiomic phenotype compared with patients with stable or no CAD. The radiomics model based on the best performing features achieved better results in discrimination of AMI if comparison to PCAT attenuation-based model or clinical model. Furthermore, the PCAT radiomic profile remained stable 6 months post-AMI.

Additionally, very recent studies highlighted the properties of radiomics-based model in combination with other already known parameters (such as FAI) or in combination with different tool of imaging analysis (as for CT-fractional flow reserve) in predicting hemodynamically significant coronary stenosis: results showed that combining PCAT radiomics model with FAI or CT-FFR offers higher diagnostic performances in the identification of coronary stenosis [43, 44].

In conclusion, radiomics FRP significantly improves individual cardiac risk profile identification beyond the current state-of-the-art and discriminates patients with AMI from those with stable disease; moreover, it could be a useful diagnostic tool in detection of PCAT persistent structural changes, providing additional risk stratification in both primary and secondary prevention.

## Radiomics application in cardiac magnetic resonance (CMR)

CMR is the optimal imaging modality for noninvasively analyzing myocardial structure and function, playing an essential role in clinical practice: structural or functional heart and vascular abnormalities, alterations of blood hemodynamics and valvular diseases, perfusion, and coronary microvascular changes can be assessed with this imaging modality. Moreover, CMR offers a unique capability in myocardial tissue characterization among alternative imaging modalities [45].

However, sometimes qualitative descriptors and basic geometric quantifiers, based on intrinsic features of different tissues, are often not sufficient to detect the gap between disease and healthy tissue or to distinguish between morphologically similar but clinically different diseases [46]. Consequently, much of the information obtained from CMR images cannot be fully exploited, in particular in case of morphologically similar but clinically different conditions [47]. Such limitations are tangible in the differential diagnosis between athletic cardiac remodeling and dilated cardiomyopathy (DCM): the first condition is a physiological adaption in response to athletic training, while the second condition is due to micro- and macrostructural pathological modification of the myocardium [48, 49]. The application of radiomics principles to CMR may help to achieve proper clinical management and to improve patient prognosis. The aim of this section is to review the current radiomics applications in CMR.

### Radiomics in CMR evaluation of structural heart disease

Cardiomyopathies are a group of primary pathologies due to a congenital or acquired structural alteration of the myocardium. According to the specific defect, four main forms are distinguished, hypertrophic cardiomyopathy (HCM), DCM, restrictive cardiomyopathy, and arrhythmogenic right ventricular dysplasia (ARVD) [50].

HCM is the most common form, with an incidence of 1/500, is characterized by generalized cardiac hypertrophy, mostly of the septum, myofibrillar disarray, and it is often cause of SCD (sudden cardiac death) in young people. HCM patients usually show typical ventricular alterations distinguishing it from other forms of cardiac hypertrophy, such as hypertensive heart disease: asymmetric distribution of hypertrophy, the presence of systolic anterior motion, and patchy LGE. However, sometimes, especially in the diffuse variants or in early stage of HCM, differentiating these two forms of ventricular myocardial hypertrophy may not be easy [51, 52].

With regard to HCM, several studies underline how radiomics represents a promising radiological tool, providing a helpful support in differential diagnosis with healthy patients and hypertensive heart disease [53].

**Fig. 3:**
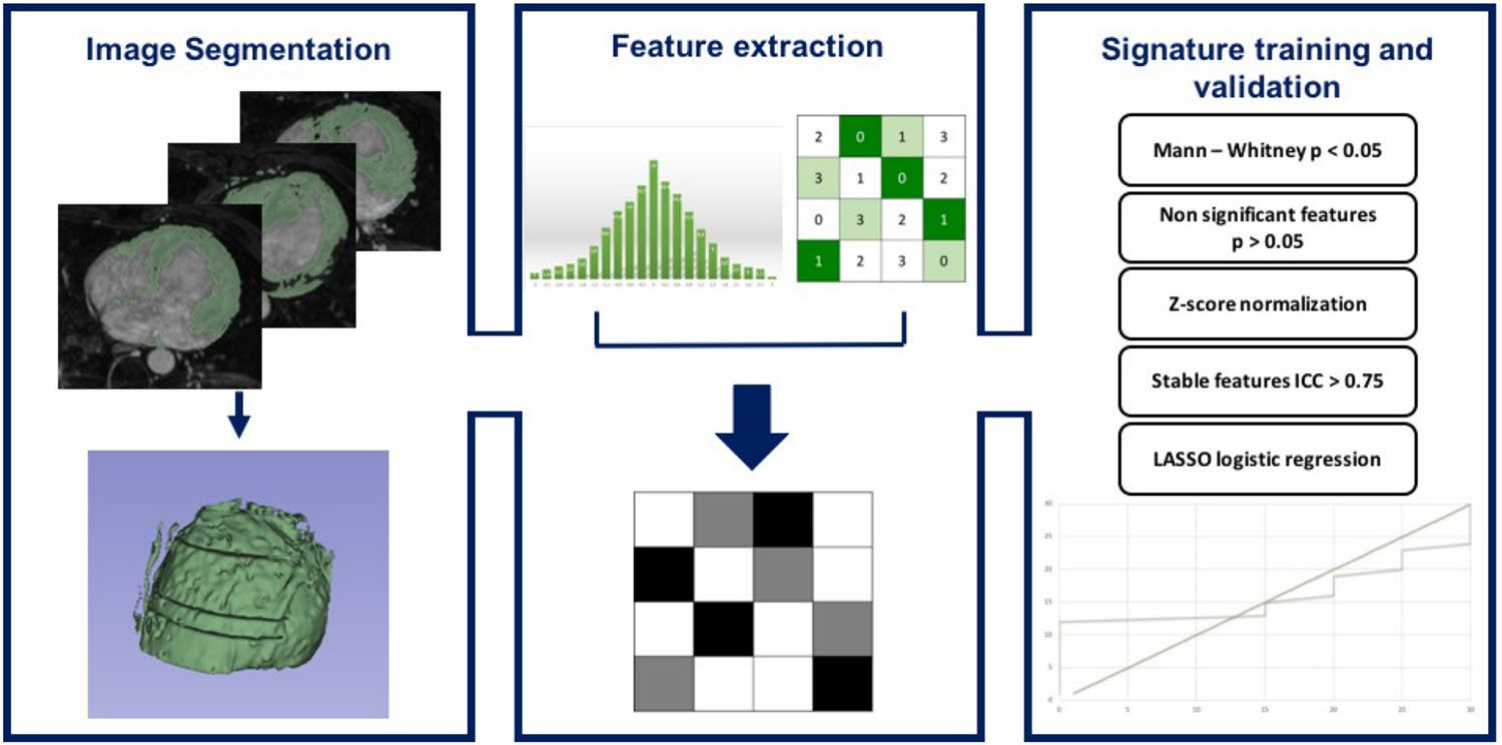
LV myocardial thickness CMR radiomics workflow. Image segmentation: myocardial manually segmented on axial MRI slice; feature extraction: myocardial thickness, texture, and integration data; statistical analysis: combination of radiomic features with clinical data to obtain predictive model. Left ventricle (LV); cardiac magnetic resonance (CMR)

Among these, Baeßler et al. [54] investigated the effectiveness of texture analysis in distinguishing hidden HCM myocardial tissue alterations compared to structural characteristics of healthy heart. Myofibrillar disorder and myocardium fibrotic remodeling in HCM patients causes signal abnormalities and inhomogeneity, usually visible on late gadolinium enhancement (LGE) sequences, but undetectable on pre-contrast T1 or T2-weighted images when performing a pure visual analysis [55]; myocardial another frequent cause of inhomogeneity be the edema which, above all in HCM, is associated with electrical instability [56]. This retrospective study included 62 patients, 32 patients with known HCM and 30 controls; HCM patients were also divided in two subgroups according to the presence (LGE +) or absence (LGE -) of LGE. TA was applied to left ventricular regions of interest (ROI), drawn on T1-weighted, non-contrast, short-axis images (Fig. 3). Results showed that four specific radiomics features resulted promising in revealing significative differences between the two groups: *GLevNonU* (gray-level non-uniformity), *WavEnLL* (energy of wavelet coefficients in low-frequency sub-band), *Fraction*, and *Sum Average*. In particular, *GLevNonU* with a cutoff value of 46 achieved the highest diagnostic performance, with 94% sensitivity and 90% specificity. *GLevNonU* showed also significant differences between LGE—patients and healthy controls, with 100% sensitivity and 90% specificity. Thus, TA on T1-weighted non-contrast images could allow the detection of myocardial tissue alterations in the HCM setting with excellent accuracy, providing new parameters for a non-contrast assessment of myocardial structure alterations, as an alternative to LGE [55, 57, 58].

Another useful CMR imaging tool is the parametric mapping, such as T1, T2, T2*, and ECV mapping. It provides a quantitative evaluation of heart relaxation times, high spatial information, and good detection of diffuse disease, usually hidden on conventional CMR [59].

Differential diagnosis between hypertensive heart disease (HHD) and HCM is not always straightforward, since both diseases are characterized by left ventricular wall thickening and diffuse fibrosis, mostly localized in the septum in HCM patients; mean native-T1 values are conventionally considered as a surrogate for fibrosis. Radiomics analysis of native T1 images has been proven effective in discriminating between HHD and HCM patients, providing incremental value over global native T1 mapping through the detection of fibrosis spatial distribution differences [49].

### Radiomics in CMR evaluation of ischemic heart disease (IHD)

Despite therapeutic advances, myocardial infarction still has an acute phase mortality about 10%, with a subsequent mortality rate of 10% in the first year of follow-up [60].

A peculiar characteristic of myocardial infarction is the segmental alteration of ventricular mechanics: the replacement of muscle tissue with non-contractile fibrous tissue is a chronic reparative response to acute myocardial infarction, involved in the process of cardiac remodeling after an acute event, and it determines regional changes in the ventricular function [61]; the size of the necrotic area determines a spectrum of pathophysiological situations characterized by different degrees of hemodynamic alterations, from ejection fraction reduction to heart failure, and with a different clinical presentation, from exercise dyspnea to death. This highlights the importance of detecting and characterizing the myocardial scar to guide intensive secondary prevention [62].

CMR is the reference standard technique for evaluating myocardial structural changes in IHD; the two most common sequences include cine-MRI sequences, essential for the evaluation of contractile function, ventricular volumes and ejection fraction, and LGE sequences, for quantifying any residual scar area [63, 64].

The quantification of the infarct area with the LGE technique is mandatory to establish the myocardial viability that represents the possibility of myocardial function restore after revascularization [65, 66]; in case of transmural extent of infarction, viability is absent. On the other side, the so-called “remote” areas are usually defined as the residual healthy myocardium, far from the infarct zone [67–71]. LGE sequences have many intrinsic limitations, above all the need of contrast administration and the prolongation of the exam: some patients, especially if hemodynamically unstable, are not able to tolerate prolonged studies or cannot be injected with gadolinium.

Patients with CAD have a high prevalence of coexisting chronic kidney disease [72], requiring alternative methods to detect myocardial scar without gadolinium administration, due to their higher risk of developing nephrogenic systemic sclerosis [73]: T1 mapping is the technique that has demonstrated more advantages in revealing myocardial scar without gadolinium administration, although its usefulness is still limited at the moment [74].

In that scenario, Larroza et al. [75] aimed at investigating CMR radiomic signature in the detection of the differences between non-viable and remote segments without gadolinium administration, based on the assumption that non-viable segments would show a different heterogeneity of the gray level compared to the remote segments. Due to the temporal factor of cine-MRI, TA was applied on both static images (2D, dimension) and cine images (2D + t, temporal factor). Features from fist to higher order were extracted from myocardial ROI (Video 1S, Supplementary Material).

Best results were obtained with 2D + t LBP (local binary patterns) features, which showed high diagnostic performance in identification of non-viable, viable, and remote segments compared with LGE evaluation.

These results confirmed what had already emerged in the study by Shriki et al. [76]: chemical displacement artifacts, detectable in cine-MRI, are indicative of chronic myocardial infarction. However, the radiomic TA application explored by Larroza et al. offered an objective quantitative way to evaluate the myocardial changes in IHD; the common limitation of prior studies was the pure visual image assessment of cine-MRI sequences, which might be challenging in clinical setting and severely affected by inter-reader variability.

Larroza et al. [77] proposed also a different CMR radiomics approach in order to differentiate AMI from chronic myocardial infarction (CMI), starting from the observation that there is no difference in the hyperintensity of the infarcted myocardium on LGE as for the age of the ischemic event [78]: their discrimination is clinically relevant when both entities coexist, since a wrong diagnosis could complicate management and treatment planning.

The idea behind the study was that the presence of edema and fibrosis characterized the most important corresponding pathological changes of heart structure in AMI and CMI, respectively; these alterations affect the gray levels of myocardium but are imperceptible to visual evaluation.

This retrospective study was performed on 44 patients, equally distributed between AMI and CMI, demonstrating that the radiomics model, based on a subset of specific features (support vector machine, SVM, with polynomial kernel), had the potential to differentiate AMI from CMI in both LGE and cine-MRI analysis, revealing that TA can be useful also on standard cine sequences, in which the infarction detection is challenging in most cases.

Baessler et al. [79] further investigated the potential role of TA as a noninvasive biomarker of subacute or CMI on nonenhanced cine-MRI images. Patients who had a subacute or chronic ischemic myocardial scar based on the results of LGE were retrospectively enrolled and divided in two subgroups, based on previous work showing an association of scar size with MACE [80]: 48 patients with a small scar (less than or equal to 20% transmural extension) and 72 patients with a large scar (greater than 20% transmural extension). Sixty patients with normal cardiac MR imaging results were included as controls. TA was performed in all 180 patients on a single cine section, drawing a circular myocardial ROI. From a total of 286 texture features, separately extracted 5 independent features reached best performances: 2 first-order features (*Perc.01, Variance*), 1 from second-order (*SSumEntrp*), and 2 higher-order features (*Teta1, WavEnHH.s-3*). The most effective statistical model was obtained combining *Perc.01* and *Teta1* features: in both large scar and small scar subgroups, it was able to differentiate between normal myocardium and fibrosis with excellent accuracy, demonstrating that TA could be a valid alternative to LGE imaging.

### Radiomics in CMR-based arrhythmia risk stratification

Cardiac arrhythmia designates a group of conditions from para-physiological state to pathological one, all characterized by irregular beats. These disorders can be clinically asymptomatic or, sometimes, may be manifested as palpitations or can cause shortness of breath and syncope [81].

Arrhythmias are particularly common in the acute phase of IMA (70–90% of cases), so much that are widely considered part of the natural history of the IHD [82]. Pathophysiological cellular changes after heart attack generally lead to a lengthening of refractory time of some myocardiocytes and a slowdown of the electrical conduction in others, generating the ideal substrate for the triggering of arrhythmic events [83].

Patients with previous AMI can be divided into high-arrhythmic risk and low-arrhythmic risk; the differential diagnosis between them is crucial for an optimal prevention of SCD since only the high-risk group has been shown to benefit from the insertion of the implantable cardioverter defibrillator (ICD) [84]. Currently, markers such as left ventricular ejection fraction and myocardial scar are used to assess arrhythmic risk. With regard to myocardial scar, the two main characteristics that play a role in defining arrhythmic risk in patients with IHD are scar size and its inhomogeneity [85].

Kotu et al. [86] tested radiomics as a tool to distinguish high- and low-risk patients by combining functional, intensity, and texture features with k-nearest neighbor (k-NN), support vector machine (SVM), decision tree and random forest classification on 54 patients (20 high risk, 34 low risk) by comparing TA applied to LGE images and conventional classification method based on left ventricular ejection fraction and myocardial scar size are.

Results showed that the radiomic-based model was comparable to the conventional model, suggesting that it could be included in future arrhythmic risk stratification or management.

Arrhythmias can be also the consequence of structural pathologies of the myocardium altering the normal cardiac electrical conduction, as frequently occur in HCM patients.

Amano et al. [87] studied the relationship between texture features extracted from LGE regions and history of ventricular tachyarrhythmias in 23 HCM patients. Among more than 100 features extracted, only 1 texture feature (*entropy LL*) reached promising results in distinguishing patients with and without a history of ventricular tachyarrhythmias (AUC 0.72), even if its performances were still lower than the visual evaluation of LGE extension (AUC 0.96).

Finally, arrhythmia can also be the result of myocarditis, an inflammatory process affecting the myocardium typically associated with viral or bacterial infections; although in some cases it is possible a complete recovery from the disease, permanent structural alterations can follow the phase of maximum acuity and compromised cardiac function and electrical conduction.

An early diagnosis remains a crucial point for an optimal therapeutic management, and CMR has now taken the place of biopsy, thanks to its less invasiveness [88].

A recent analysis by Baessler et al. [89] conducted on 39 patients with infarct-like presentation and clinical suspicion of acute myocarditis showed that the application of TA to T2 mapping sequences resulted in high diagnostic performance: a model obtained combining T2 run-length plot non-uniformity and gray non-uniformity features reached 89% sensitivity and 92% specificity in detection of myocarditis, suggesting a promising role of TA also in field of inflammatory heart disease.

## Conclusion

Radiomics is an innovative quantitative image analysis technique with the potential to improve diagnostic and predictive capabilities of both CT and MRI examinations, providing unique information about tissue pathophysiology and with the potential to overcome the limit of subjective imaging evaluation.

Although there are still many unresolved technical challenges, the current combination of big-data availability and high computational power allows us to consider these challenges as achievable.

On the other side, radiomics tools and applications are fields in constant development, not free from critical issues that should be faced and resolved. To date, the major limitation of existing studies is the absence of external validation and lack of standardization of the proposed models. Therefore, it is necessary to build models on large, high-quality training datasets, and further multicentric studies are necessary to keep testing and validating the radiomics approach, in order to implement radiomics in clinical use [90]. In this regard, guidelines in field of radiomic were recently issued by the European Society of Radiology, aiming at standardizing the radiomic workflow, as this should be the first step to be implemented in order integrate radiomics data in clinical routine [91, 92].

In the specific field of cardiovascular imaging, radiomics could be considered as a complementary tool for the clinicians, allowing them to combine clinical and quantitative data and to take another step forward toward personalized medicine.

## Supplementary Information

Below is the link to the electronic supplementary material.

Video 1S: Myocardial Volumetric Segmentation. Example of volumetric segmentation of left ventricle using a free open-source software (3D Slicer), applied to a 3D whole heart sequence: left ventricle wall was semi-automatically selected through threshold modulation of voxel intensity values and the 3D-volume was extracted, after automatic exclusion of extra-cardiac tissues (MP4 5982 KB)

## Abbreviations

AMI: Acute myocardial infarction
ARVD: Arrhythmogenic right ventricular dysplasia
AUC: Area under the curve
CAD: Coronary artery disease
CCTA: Coronary computed tomography angiography
CMI: Chronic myocardial infarction
CMR: Cardiac magnetic resonance
DCM: Dilated cardiomyopathy
HCM: Hypertrophy cardiomyopathy
HHD: Hypertensive heart disease
ICD: Implantable cardioverter defibrillator
IHD: Ischemic heart disease
IVUS: Intravascular ultrasound
LGE: Late gadolinium enhancement
LVWT: Left ventricular wall thickness
MACE: Major adverse cardiovascular events
NPV: Negative predictive value
NRS: Napkin-ring sign
OCT: Optical coherence tomography
PCAT: Pericoronary adipose tissue
SCD: Sudden cardiac death
ROI: Region of interest
TA: Texture analysis
